# Saved by Menorrhagia: Spontaneous Coronary Artery Dissection Complicated With a Left Ventricular Thrombus

**DOI:** 10.7759/cureus.47623

**Published:** 2023-10-25

**Authors:** Amit Ramrattan, Kevin Maraj, Raelene Allen

**Affiliations:** 1 Internal Medicine, Port-of-Spain General Hospital, Port-of-Spain, TTO

**Keywords:** acute coronary syndrome, st-elevation myocardial infarction (stemi), coronary artery angiogram, ventricular thrombus, spontaneous coronary dissection

## Abstract

Spontaneous Coronary Artery Dissection (SCAD) is a non-atherosclerotic, non-iatrogenic cause of Acute Coronary Syndrome (ACS) resulting in a tear in the coronary artery wall with subsequent myocardial infarction. Though rare, it has been increasingly recognized, especially in young women. This is a case report on SCAD where, fortuitously, the patient did not receive standard tissue plasminogen activator (TPA) on presentation of suspected ST-elevation Myocardial Infarction (STEMI). Subsequent investigations revealed a left ventricular thrombus on echocardiogram and SCAD on coronary angiogram.

## Introduction

Since its first reported case in 1931 [1], SCAD has grown much interest over the years with a diagnosis shift from autopsy series to coronary angiogram. It is a rare cause of ACS, defined as non-atherosclerotic, non-iatrogenic, non-traumatic separation of the coronary artery wall [2]. SCAD originates either from an intimal tear that dissects into the arterial wall or from spontaneous rupture of the vasa vasorum. Both mechanisms lead to intramural bleeding of an epicardial coronary artery wall, where the resultant intramural hematoma compresses the artery lumen, resulting in ischemia or infarction [3]. Despite the growing awareness of the condition, there is still a paucity of data with regard to acute and long-term management. However, the consensus from the American Heart Association (AHA), American College of Cardiology (ACC), and European Society of Cardiology (ESC) favors a conservative approach as opposed to coronary revascularization (percutaneous intervention or coronary artery bypass graft surgery (CABG)) [4-6]. The presenting case is an extremely rare occurrence of a left ventricular thrombus complication from SCAD. In a resource-limited setting of the Port-of-Spain General Hospital (POSGH), where thrombolysis has been the standard of care for patients presenting with a STEMI, this patient who presented as an ACS did not receive such treatment and was subsequently found to have a ventricular thrombus and SCAD.

## Case presentation

A 31-year-old female with a past medical history of morbid obesity with a body mass index of 38kg/m^2^, gastroesophageal reflux disease, and anemia secondary to menorrhagia presented to the emergency department with sudden onset of light-headedness, retrosternal chest pain that radiated to the left shoulder, and nausea in addition to two non-bilious vomiting episodes. There was no history of oral contraception usage, no family history of coronary artery disease, connective tissue or systemic inflammatory disease. She admitted to a stressful life having to work two jobs and provide care for three young children as her spouse had recently died. The patient had a three-pack-year cigarette smoking history but denied the use of recreational substances.

Admission vital signs revealed a blood pressure of 125/68 mmHg, pulse rate of 80 beats per minute, respiratory rate of 22 breaths per minute, and pulse oximetry of 98% on room air. She appeared to be comfortable but complained of light-headedness. The rest of the patient’s physical examination was unremarkable and there was no evidence of orthostatic hypotension.

Laboratory investigations yielded a hemoglobin of 8.1 g/dl, mean corpuscular volume of 90.6 fL, white blood cell count of 14.5 x10^3^/µL, platelet count of 381 x10^9^/µL, international normalized ratio 1.26 and erythrocyte sedimentation rate of 7.0 mm/hour. The patient’s renal function, liver function, and thyroid function tests were normal. The lipid profile was within the normal range. Complement factors three and four were normal but testing for antinuclear antibodies was unavailable then. An HIV rapid report was negative with a non-reactive venereal disease research laboratory (VDRL) test. Highly sensitive Troponin T at more than three hours post onset of chest pain was 334 pg/ml which on repeating yielded a result of 89 pg/ml (normal range 12.7-24.9 pg/ml). An electrocardiogram obtained on the admission date showed ST segment elevation in the precordial leads V3 to V6 and a chest radiograph done on admission showed a normal cardiac silhouette with clear lung fields.

The initial management of the patient was for an anterolateral wall ST-elevation myocardial infarction. The patient’s history of menorrhagia and anemia prohibited the use of tissue plasminogen activator. In the emergency department, the patient was given orally high-dose aspirin 324mg, clopidogrel 300mg, along with subcutaneous (SC) enoxaparin 60mg. The management on ward admission included dual antiplatelet therapy with aspirin 81mg once daily, clopidogrel 75mg once daily, enoxaparin 80mg SC twice daily, carvedilol 3.125mg twice daily, trimetazidine modified release 35mg twice daily, and enalapril 2.5mg twice daily. 

**Figure 1:**
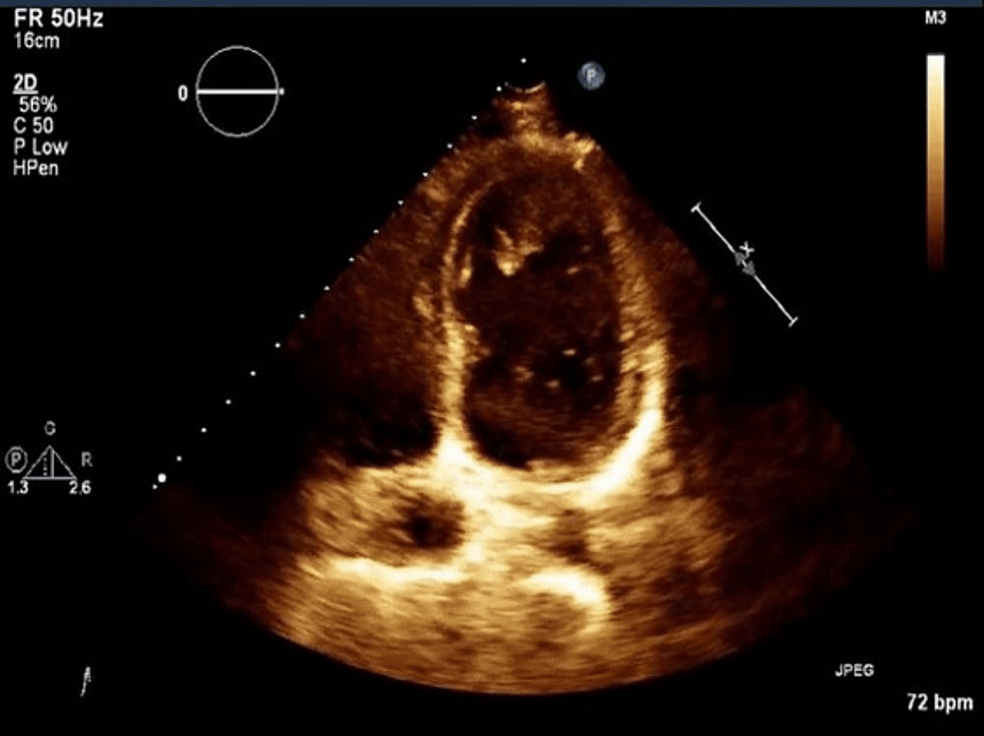
Echocardiogram showing an apical thrombus The thrombus dimension was 29mm x 14mm in the patient with an anterolateral wall ST-elevation myocardial infarction (STEMI).

On day five of her hospital stay, due to limitations in cardiology services, an echocardiogram was done (Figure 1) which revealed a myocardial infarction involving the apical left ventricular segment along with a large apical thrombus at 29mm by 14mm in size. The calculated ejection fraction was 74.4% by Simpson's method.

**Figure 2:**
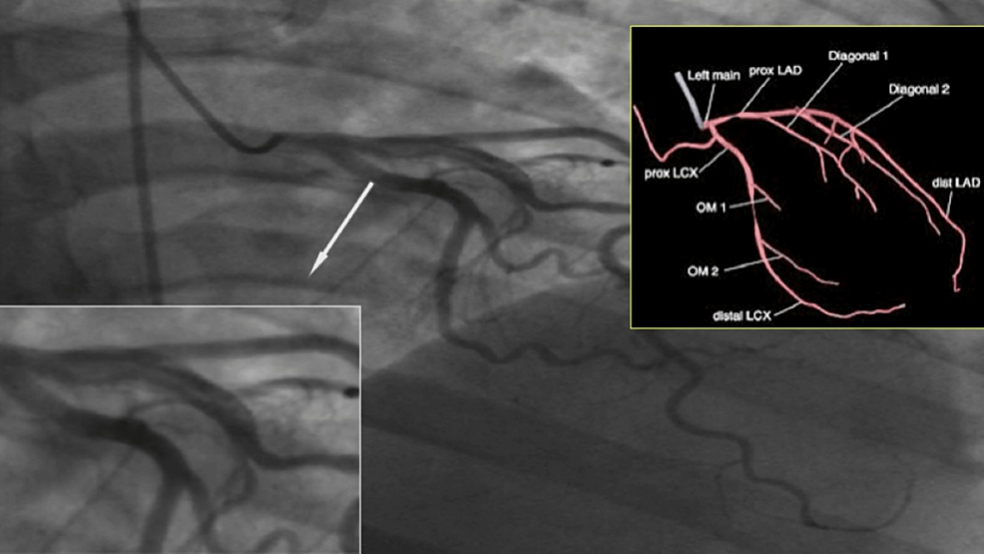
Coronary angiogram with LAD dissection extending from the ostium to the 1st diagonal artery LAD: Left anterior descending artery

Warfarin was commenced post echocardiogram and a therapeutic INR range between 2-3 was achieved. The patient remained hemodynamically stable while hospitalized and on day 15 had a coronary angiogram done through a private institution (Figure 2).

The angiogram demonstrated a left anterior descending coronary artery Type 2 dissection extending from the LAD ostium to the first diagonal artery. There were no stenotic lesions or thrombus noted and anterograde flow was normal. The left main coronary and circumflex arteries had no stenotic lesions. The right coronary artery opacified well and showed no stenotic lesions throughout the entire vessel. She was discharged on the day of the coronary angiogram on warfarin, carvedilol, and rosuvastatin. A follow-up plan was made to attend the medical clinic with further monitoring of the anticoagulation therapy and clinical status of the patient.

## Discussion

The true prevalence of SCAD is unknown as it is largely underdiagnosed. However, studies show that it accounts for 1%-4% of ACS cases [7], 15%-20% of peripartum ACS cases [8], and up to 35% of ACS cases in women under the age of 60 [9]. SCAD has also been reported to have occurred after precipitating events such as intense exercise, illicit drug use, emotional turmoil, and other physical stressors [5]. Conditions associated with SCAD include fibromuscular dysplasia, chronic systemic inflammatory diseases, connective tissue disorders, pregnancy, and hypothyroidism [5,10].

SCAD is a diagnosis that must be considered in a young patient presenting as ACS, especially if the patient is a female and devoid of risk factors for atherosclerosis. Pain is typically the presenting complaint in more than 95% of patients [6] with STEMI presentation ranging between 25%-75% [11], and the remainder having non-ST elevation myocardial infarction (NSTEMI), ischemic changes on electrocardiogram and elevated cardiac biomarkers. Coronary angiography is the gold standard for diagnosis [12]; however, it comes at the risk of propagating the dissection. SCAD has three angiographic classifications [13]. Type 2 SCAD, which is most common (70%), is characterized by diffuse smooth stenoses that are typically greater than 20mm. Type 1 (25%) is a contrast staining of the arterial wall that gives the appearance of multiple radiolucent lumens, and type 3 (5%) is where there is a focal or tubular stenosis, typically less than 20mm and gives the appearance of atherosclerosis. Although any coronary artery can be affected, the left anterior descending artery (LAD) is commonly affected in 32-46% of cases [4]. The mentioned risk factors and presentation of SCAD are reflected in this case as the patient is a young female, devoid of atherosclerosis risk factors, has emotional and physical stressors with the recent death of her spouse and multiple jobs, who presented as a STEMI and was subsequently found to have a type 2 SCAD.

Management of SCAD is largely conservative, as studies have demonstrated that percutaneous coronary intervention (PCI) in this setting had poor outcomes and high complication rates [14]. Furthermore, where revascularization was needed either with PCI or CABG, neither intervention was protective against recurrent dissection [15,16]. In the absence of randomized controlled trials, dual antiplatelet therapy (DAPT) has been recommended in the management of SCAD and has been shown to be safe [8,17]. The role of DAPT is to maintain lumen patency and minimize thrombus burden. Beta-blockers, just as their role in minimizing the propagation in acute aortic dissection, are believed to have the same effect in SCAD as well as possibly reducing the risk of recurrence [9]. Thrombolysis is contraindicated as it can lead to coronary artery rupture or tamponade [18,19]. Fortuitously, in this case, the patient’s anaemia prohibited using a thrombolytic.

SCAD complicated with a left ventricular thrombus is extremely rare with few case reports documented. This patient developed this complication that warranted the use of warfarin to decrease the risk of embolization and stroke. One similar case report by Joo and colleagues [20] illustrated the successful treatment of SCAD with a left ventricular thrombus with antiplatelets and anticoagulation.

## Conclusions

In a resource-limited setting where a coronary angiogram is not readily available, SCAD is an important differential to consider in a patient presenting as an ACS. The wrong management strategy may have dire consequences if the condition is not sought. Therefore, clinicians must second guess a young ACS patient bereft of atherosclerosis risk factors. With the limited evidence available, randomized controlled trials are needed to validate current practice as the prevalence is expected to grow with increasing clinician awareness and advancements in angiographic techniques.

## References

[1] Pretty HC (1931). Dissecting aneurism of coronary artery in a woman aged 42. Br Med J.

[2] Saw J, Mancini GB, Humphries KH (2016). Contemporary review on spontaneous coronary artery dissection. J Am Coll Cardiol.

[3] Saw J (2013). Spontaneous coronary artery dissection. Can J Cardiol.

[4] Hayes SN, Kim ES, Saw J (2018). Spontaneous coronary artery dissection: current state of the science: a scientific statement from the American Heart Association. Circulation.

[5] Hayes SN, Tweet MS, Adlam D, Kim ES, Gulati R, Price JE, Rose CH (2020). Spontaneous coronary artery dissection: JACC state-of-the-art review. J Am Coll Cardiol.

[6] Adlam D, Alfonso F, Maas A, Vrints C (2018). European Society of Cardiology, acute cardiovascular care association, SCAD study group: a position paper on spontaneous coronary artery dissection. Eur Heart J.

[7] Gad MM, Mahmoud AN, Saad AM (2020). Incidence, clinical presentation, and causes of 30-day readmission following hospitalization with spontaneous coronary artery dissection. JACC Cardiovasc Interv.

[8] Kim ES (2020). Spontaneous coronary-artery dissection. N Engl J Med.

[9] Saw J, Humphries K, Aymong E, Sedlak T, Prakash R, Starovoytov A, Mancini GB (2017). Spontaneous coronary artery dissection: clinical outcomes and risk of recurrence. J Am Coll Cardiol.

[10] Motreff P, Malcles G, Combaret N (2017). How and when to suspect spontaneous coronary artery dissection: novel insights from a single-centre series on prevalence and angiographic appearance. EuroIntervention.

[11] Krittanawong C, Gulati R, Eitzman D, Jneid H (2021). Revascularization in patients with spontaneous coronary artery dissection: where are we now?. J Am Heart Assoc.

[12] Macaya F, Salazar CH, Pérez-Vizcayno MJ (2019). Feasibility and safety of intracoronary imaging for diagnosing spontaneous coronary artery dissection. JACC Cardiovasc Imaging.

[13] Saw J (2014). Coronary angiogram classification of spontaneous coronary artery dissection. Catheter Cardiovasc Interv.

[14] Tweet MS, Hayes SN, Pitta SR (2012). Clinical features, management, and prognosis of spontaneous coronary artery dissection. Circulation.

[15] Tweet MS, Eleid MF, Best PJ (2014). Spontaneous coronary artery dissection: revascularization versus conservative therapy. Circ Cardiovasc Interv.

[16] Lettieri C, Zavalloni D, Rossini R (2015). Management and long-term prognosis of spontaneous coronary artery dissection. Am J Cardiol.

[17] Rogowski S, Maeder MT, Weilenmann D (2017). Spontaneous coronary artery dissection: angiographic follow-up and long-term clinical outcome in a predominantly medically treated population. Catheter Cardiovasc Interv.

[18] Zupan I, Noc M, Trinkaus D, Popovic M (2001). Double vessel extension of spontaneous left main coronary artery dissection in young women treated with thrombolytics. Catheter Cardiovasc Interv.

[19] Shamloo BK, Chintala RS, Nasur A, Ghazvini M, Shariat P, Diggs JA, Singh SN (2010). Spontaneous coronary artery dissection: aggressive vs. conservative therapy. J Invasive Cardiol.

[20] Joo JH, Caldera AE, Divakaran VG (2019). Spontaneous coronary artery dissection with left ventricular thrombus. Proc (Bayl Univ Med Cent).

